# Separation between mothers and newborns directly after birth: mothers’ experiences of separation – a qualitative analysis from a cohort study

**DOI:** 10.1136/archdischild-2025-329190

**Published:** 2025-11-12

**Authors:** Emilia Biskop, Ylva Thernström Blomqvist, Barbro Diderholm, Alkistis Skalkidou, Maria Grandahl

**Affiliations:** 1 Department of Women’s and Children’s Health, Uppsala universitet Medicinska fakulteten, Uppsala, Sweden

**Keywords:** Neonatology, Intensive Care Units, Neonatal, Infant Welfare

## Abstract

**Objective:**

The first hour after birth is a sensitive period for the mother–newborn dyad and, according to evidence-based routines, should be respected and protected. However, a gap exists between the strive for zero separation and current practices. Therefore, our aim was to investigate mothers’ experiences of mother–infant separation directly after birth.

**Design, setting and participants:**

A qualitative study based on open-ended questions from a survey. The sample was drawn from an ongoing national cohort study (Mom2B, N=∼9000). Data was extracted from responses of participants (n=441) during the period 2023–2025. We included mothers who had been separated from their newborns directly after birth and who had completed the open-ended questions about separation and their related experiences. Thematic analysis was conducted.

**Results:**

One overarching theme emerged: ‘Separation, an experience beyond the mother’s control’. This theme referred to the healthcare system being organised in such a way that two individuals in need of care from different units could not stay together, even when the individuals were mothers and their newborn. The mothers and their newborns were often separated, even if only one of them needed care, due to existing routines and organisation. Mothers questioned the reasons for separation and described it as anxiety-inducing and traumatic.

**Conclusion:**

The mothers’ experiences and reasons for separation highlight structural obstacles within a healthcare system that need to be addressed to minimise the significant burden of such separation.

**WHAT IS ALREADY KNOWN ON THIS TOPIC**

- There is a gap between the strive for zero separation and current practices in the care of newborns and their mothers.

**WHAT THIS STUDY ADDS**

- Mothers and newborns were often separated, even if only one of them needed care, due to existing routines or infrastructure. Separation was described as terrible and painful.

**HOW THIS STUDY MIGHT AFFECT RESEARCH, PRACTICE OR POLICY**

- Mothers’ experiences and reasons for separation highlight structural obstacles within healthcare that need to be addressed to minimise the significant burden of such separation.
- Healthcare needs to change routines. Separation should be avoided through uninterrupted skin-to-skin contact and through caring for the mother and the newborn together in couplet care, that is, to implement organisation that supports zero separation.

## Introduction

 The first hour after birth is a sensitive period for both the mother and the newborn and should be respected and protected by evidence-based routines.^1^ The WHO recommends that immediate, continuous, uninterrupted skin-to-skin care should be standard care for all mothers and infants after all modes of delivery, unless the infant is critically sick.^2^ The importance of zero separation for optimal physiological outcomes is well described. This is a concept where the newborn is cared for skin-to-skin with one of the parents and one parent is always present with the newborn.^3 4^ Furthermore, mother–newborn separation can be traumatic, affecting the whole family for a long time.^4 5^ Despite this, there appears to be a gap between the theoretical knowledge of the effects of separation and the clinical practices currently used in the care of newborns and their mothers.^6^

What happens during the newborn’s first hours is mainly controlled by healthcare staff. Routines and care practices can both promote and hinder parent–newborn closeness. Closeness refers mainly to skin-to-skin care, which is considered routine in Sweden. According to Swedish national guidelines, routine care, such as measuring the newborn, should be postponed until after the infant has been cared for skin-to-skin for 2 hours. Whenever possible, medical procedures such as respiratory support or blood sampling should be conducted during skin-to-skin contact.^7^ Still, mother–newborn separation during the first hours after birth is common, mainly due to conflicting routines.^2 8 9^ Mother–newborn couplet care is a concept where both the mother and the newborn receive care from birth to discharge at the same ward, irrespective of whether one or both are in need of specialised care.^10^ To date, Swedish national guidelines exist for this, but there has not yet been any systematic follow-up regarding adherence. To reduce separation after birth, various changes in working methods and caregiver attitudes are needed, including increased knowledge, education, training and improvements in environmental and organisational structures.^2 9 10^ Healthcare staff support couplet care but highlight the need for close collaboration between different units.^11^

Separation can involve significant emotional strain, even if the newborn is not seriously ill.^12^ Despite feeling anxious, many mothers wish to keep their newborns with them and practise skin-to-skin care immediately after caesarean birth.^13^ Furthermore, early mother–newborn closeness after birth is associated with a more positive birth experience for the mother, especially for those delivering by caesarean section.^14^

We aimed to investigate mothers’ experiences of mother–newborn separation directly after birth.

## Methods

### Design and setting

This qualitative study is reported according to COREQ (Consolidated criteria for Reporting Qualitative research) checklist (online supplemental file 1). It is part of an ongoing national mobile application-based cohort (mom2b.se, N=∼9000), available for download to anyone who is pregnant or up to 3 months postpartum, over the age of 18, understands Swedish and owns a smartphone. Data are collected from the time of enrolment until 1 year after birth. The overall aim is to use digital phenotyping data from the Mom2B smartphone application to develop models to predict women at high risk of mental and somatic complications. The population represents all 21 regions in Sweden and the majority have a university degree. More information is available at https://mom2b.se, and in the following publications including the study protocol.^15–17^

### Study population

Deidentified data were extracted from the Mom2B database from August 2023 to April 2025. Participants in the present study were eligible for inclusion if they had experienced separation from their newborns after birth (Yes/No), and had completed the open-ended questions regarding separation.

### Data collection

The Mom2B study collects data from self-reported questionnaires, voice recordings and digital behavioural data.^15^ A list of self-reported questions is presented via the mobile application at preselected times during pregnancy and postpartum. For the current study, the related open-ended questions included: ‘Were you separated from your newborn in connection with the delivery?’, ‘How long were you separated? Enter your answer in minutes’, ‘Why were you and your newborn separated?’ ‘Was your newborn admitted to a neonatal unit? For how long? Enter your answer in days’, ‘Why was your newborn admitted to a neonatal unit?’ and ‘Comment on the care received in the neonatal unit’. The following variables were also included: demographic background variables of the mother, details on pregnancy variables, mode of childbirth and the newborn’s characteristics (table 1).

**Table 1:** Background characteristics of the study population, N=441 (the number of missing values varies across rows)

Characteristics of study sample		Study sample Mean (range)	Study sample Frequency (%)
Mother	Age, years	32 (21–45)	
	Born outside Sweden		33 (8)
	University or college education		322 (76)
	Previous children		152 (36)
	In a relationship		414 (98)
Pregnancy	Level of pregnancy planning		
	Highly planned		249 (58)
	Quite planned		88 (21)
	Neither planned nor unplanned		39 (9)
	Quite unplanned		28 (6)
	Highly unplanned		24 (6)
	Multiple pregnancy		13 (4)
Mode of delivery	Vaginal		170 (45.6)
	Vacuum extraction		37 (9.9)
	Planned caesarean		36 (9.7)
	Acute caesarean		130 (34.9)
Newborn infant	Cared for in a neonatal unit		126 (28.8)
	Days cared for in a neonatal unit	11.7 (0.25–104)	
Mother and infant	Separated from each other, minutes	241 (2–5600)	

### Data analysis

Demographic background data were analysed with descriptive statistics using SPSS V.28.0 statistical software (SPSS). The open-ended questions were analysed with thematic analysis using an inductive approach.^18^ The analytical process followed six phases based on the study’s aim.

1. The material was read several times by the authors (EB, YTB and MG) to become acquainted with the data, and initial thoughts were noted.
2. Codes were systematically produced throughout the data. These codes characterised features of the data material.
3. The codes were analysed by grouping them into possible subthemes, themes and one overarching theme.
4. The themes were refined and revised.
5. The themes were defined, and the main features of each theme were established.
6. The finalisation of the manuscript.

## Results

A total of 2081 mothers had answered the questions about separation, and 441 (21%) had been separated from their newborns and were included in the analysis. Of those, 126 reported experiences with neonatal care. The majority were Swedish born, held a university degree and had a mean age of 32 years. The mean time for separation was 241 min, ranging from 2 to 5600 min (table 1).

Based on the analysis (table 2), one overarching theme was constructed: *‘*Separation, an experience beyond the mother’s control*’*. This theme highlights how the healthcare system’s organisation does not allow the mother and newborn, both in need of care, to stay together. Instead of caring for the mother and newborn in the same unit and allowing specialised healthcare staff to collaborate, they were separated. Additionally, three themes and four subthemes were constructed.

**Table 2:** Overview of themes and subthemes

Overarching theme	Themes	Subthemes
Separation, an experience beyond the mother’s control	Helplessness when the newborn required medical care	Momentary distress during brief interventions
		Prolonged anxiety when advanced interventions were needed
	Feeling vulnerable and sidelined when the mother required care	Feeling excluded during caesarean section
		Experiencing powerlessness during obstetric complications
	Double burden when both the mother and newborn required care	

### Helplessness when the newborn required medical care

Separation of the mother and newborn infant could occur if the newborn required care. Newborns requiring ventilatory support directly after birth were frequently cared for in a different room from the mother. Occasionally, the other parent was present with the newborn during these interventions. Those needing care in a neonatal unit were often separated from one or both parents for some time. Not knowing what was happening to their infant was described as straining.

#### Momentary distress during brief interventions

Mothers mentioned standard procedures, such as weighing and measuring the newborn, as reasons for separation. Another reason for separation was when the newborn required ventilatory support at the birth unit or for a short period at the neonatal unit.

#### Prolonged anxiety when advanced interventions were needed

Mothers used both negative and positive terms when describing their stay in the neonatal unit. While the care of the newborn was deemed necessary and accepted, it was also found to be mentally exhausting. In cases where the newborn was cared for in an open bay unit, mothers responded that both parents found it challenging, since they could hear and see other parents struggling. Not being able to stay with their newborns around the clock was described as terrible and traumatic. Conversely, parents who could participate in caring for their newborns found the experience positive and empowering. *‘*A trauma for us parents that he needed to be cared for there, but the care itself was fantastic. Good for us that we were expected to take part in the care ie, changing nappies, tube feeding, etc*’.* (#27)

Mothers described the facilities at the neonatal unit as suboptimal, sometimes lacking parental beds so the parents could not stay in the newborn’s care space around the clock.

*‘*At night, the parents were expected to be separated from their children if they were cared for in a multi-child ward, which must be seen as the opposite of the family-centred care that they claim to be providing. This led to increased stress and insecurity’. (#68)

### Feeling vulnerable and sidelined when the mother required care

When the mother needed care, this could also result in separation. Reasons for maternal care included obstetrical laceration requiring stitching in the operating theatre, caesarean section, haemorrhage, manual removal of the placenta or pre-eclampsia. Some mothers also specified their own need for rest as a reason for separation from their newborns. *‘*More information is needed regarding the division of responsibilities between parents and healthcare staff when you can’t stay with your infant. Couplet care is also needed if the mother requires continued treatment’. (#3)

#### Feeling excluded during caesarean section

Many mothers stated that routines concerning caesarean sections led to separation. It was common for the other parent to stay with the newborn while the mother was not present. Some mothers expressed that even standard routines, such as the other parent cutting the cord in another room, were perceived as separation. In other cases, the mother was cared for in a postanaesthesia unit, preventing the newborn from being with her. Mothers believed that these routines existed due to economic reasons or bureaucracy. They felt that there were no good explanations for this separation. *‘*Post-op care unit after emergency caesarean section. Due to cost savings, the newborn may not come along*’*. (#308)

#### Experiencing powerlessness during obstetric complications

There were other reasons for shorter separations when the mother needed care. The mother may have required stitching for a vaginal tear in the operating theatre or on the birth unit, yet her newborn was unable to stay with her. This was also the case with heavy postpartum bleeding. Even in these situations, mothers were sceptical about separation. ‘He got stuck during delivery, so they had to pull him out. After he had been seen by the paediatrician, I wasn’t able to hold him because they were stitching me up and I was completely exhausted. I got to hold him on my chest three hours later. At that time, it was about surviving, but now it feels difficult because you are ‘supposed’ to have the baby on your chest as soon as possible*’*. (#22)

### Double burden when both the mother and newborn required care

Separation often occurred if both the mother and the newborn needed care.

It was challenging to be cared for in different units, as the mothers’ needs were often forgotten when they were with their newborns in the neonatal unit. The settings in the neonatal units were suboptimal for a new mother; for example, there could be difficulties accessing a bathroom or shower. Also, access to food for the parents was limited in the neonatal unit. When the communication between the healthcare staff at different units was of good quality, the mothers felt secure and involved. The mothers described that the medical information about the newborn was often of good quality, but information concerning other aspects was lacking. If communication with the healthcare staff was suboptimal, the mothers felt excluded and experienced feelings of anxiety and panic. ‘Poor communication between departments. There were no medical reasons to keep us separated, only administrative mismanagement*’*. (#98)

Mothers described that being cared for in the neonatal unit was a rough beginning to the newborn’s life. ‘Great care, but they should let both parents join. I panicked when they took him from me and forced them to drive me to him in a wheelchair. I felt like I was going to die when they took him away from me’. (#54)

## Discussion

This study has revealed mothers’ experiences of being separated from their newborns directly after birth. Not being able to stay with their infant triggered feelings of powerlessness, anxiety and stress. They perceived the healthcare system as being organised in a way that prevents individuals requiring care from different units from staying together—even when those individuals are a mother and her newborn. Separation could occur even if only one of them required treatment, due to established routines or infrastructural limitations.

Caring for a whole family together, with zero separation regardless of the kind of care they need, should be standard practice. According to our results, this is not currently the case in Sweden, despite strong evidence supporting this approach.^3 4 10^ Our findings show that mothers are sceptical about how the care is organised. The success of research implementation in healthcare depends on changes in clinician/consumer behaviour, and it is critical that an implementation strategy includes this aspect.^19^ Since there are already national recommendations advocating zero separation, it is of utmost importance that healthcare staff begin to regard mother and newborn as an inseparable unit.

This study indicates that the definition of separation differs between mothers and healthcare staff. Some perceived standard procedures, such as measuring the newborn in the same room, as a separation, even though healthcare staff might not see it that way. The mothers reported separation times from 2 to 5600 min, which suggests that even short periods apart from their newborn were perceived as significant separations. This aligns with previous findings^9^ where many midwives separated newborns from their parents when the newborns needed breathing support. While this might be considered standard procedure by healthcare staff, it could be traumatic for parents. Separation of full-term newborns from their mothers should be avoided as resuscitation can be provided at the mother’s side with the placental and cord circulation intact.^20^ Even brief separations can have significant effects and should be recognised by healthcare staff.

Our results show that when communication between different units fails, it can negatively affect the mother. They felt excluded and experienced anxiety and panic. When aiming for zero separation in an organisation that does not enable couplet care, communication between different units should be prioritised. Simple measures could have tremendous effects for the mother, who is already in a vulnerable situation. Our results reflect that mothers found the settings in the neonatal unit suboptimal. They also expressed that their needs were often forgotten when they were with their newborns in the neonatal unit. Some of these problems could be solved by improved communication between different units.

Some mothers in this study experienced separation from their newborns in connection with a caesarean section and were sceptical about it. Mothers who deliver by caesarean section want their newborns to stay with them, even though it may scare them.^12^ This aligns with our results. Furthermore, separation involves significant emotional strain; therefore, a healthy newborn should not be separated from his or her mother due to guidelines or practices. Midwives could use action items to help increase their communication with parents to check and see if they agree with separation for standard procedures. Changes in working methods, as well as environmental and organisational improvements, are needed.^2 9 10^ Additionally, targeted training is crucial to ensure quality care without separation.^21^

Finally, our results show that mothers find the neonatal unit setting to be straining. Being cared for in an open bay unit and not being able to stay with their newborn around the clock was described as terrible. Closeness between parents and newborns is often impaired during a stay in a neonatal ward, but that parents’ feelings of involvement increase if they are able to participate in the care of the newborn and are given the opportunity to stay at the unit around the clock.^22 23^

### Strengths and limitations

This study is part of the national Mom2B-cohort and provides a unique insight into mothers’ experiences of separation from their newborns after birth. The participants had answered the specific questions and thus had experience in the topic at hand, representing different settings and backgrounds. The open-ended questions and the demographic variables provided comprehensive data. However, most participants were Swedish-born with a university degree. This is unfortunately common in studies that exclude individuals who have not mastered the domestic language. One can only speculate on how the responses of these women differ from those who do not speak Swedish and/or have a low socioeconomic status. It is possible that separation is even more common in this population, as these mothers may have difficulties advocating for themselves.

Furthermore, it is possible that the results may have been affected by response bias regarding how participants defined and experienced separation. An event may be regarded as a separation by some and not by others. This is especially true for brief separations, as a short time of removal of the newborn could be experienced either as a separation or simply a check-up on the newborn. The data collection comprises self-reported questions; thus, we do not know how many participants were objectively separated. Additionally, we lack information concerning the gestational age at birth, sex or birth weight of the newborns. Therefore, while the results cannot be generalised, they are likely transferable to similar settings, offering valuable insights for all healthcare staff working with newborns and their mothers.

### Clinical implications

The findings highlight the need to eliminate unnecessary separations, even brief ones, and to promote zero separation. More healthcare facilities should enable ventilator and non-invasive therapies during skin-to-skin care. When separation is unavoidable, all parents should receive support tailored to their own experience, not the caregiver’s.

Strong evidence must be translated into practice even though changing behaviours is not simple.^17^ Implementation is most effective when the required changes are precisely known. The success of research implementation in healthcare depends on changes in clinician/consumer behaviour, and it is critical that the implementation strategy includes this aspect. This needs to be considered when constructing new guidelines and policies. International and national guidelines^2 8^ emphasise that separation should be avoided by promoting uninterrupted skin-to-skin care and couplet care. Which means that organisational practices supporting zero separation between the mother and her newborn must be implemented.

## Conclusion

Even brief separations can be distressing, as stated by the participating mothers. Healthcare staff must stay attuned to individual experiences and emotional needs. All units caring for mothers and newborns should collaborate, communicate and recognise the short- and long-term harms of separation. According to national guidelines, care should be provided without separation, which is not always the case in Sweden today. Structural barriers behind these separations must be addressed to ease their burden.

## Supplementary material

10.1136/archdischild-2025-329190 online supplemental file 1

## Data Availability

Data are available on request. Restrictions apply to the availability of these data.
